# Development of a locally advanced orthotopic prostate tumor model in rats for assessment of combined modality therapy

**DOI:** 10.3892/ijo.2013.1858

**Published:** 2013-03-19

**Authors:** VASU TUMATI, SANJEEV MATHUR, KWANG SONG, JER-TSONG HSIEH, DAWEN ZHAO, MASAYA TAKAHASHI, TIMOTHY DOBIN, LEAH GANDEE, TIMOTHY D. SOLBERG, AMYN A. HABIB, DEBABRATA SAHA

**Affiliations:** 1 Departments of Radiation Oncology, University of Texas Southwestern Medical Center, Dallas, TX 75390; 2 Urology, University of Texas Southwestern Medical Center, Dallas, TX 75390; 3 Radiology, University of Texas Southwestern Medical Center, Dallas, TX 75390; 4 Advanced Imaging, University of Texas Southwestern Medical Center, Dallas, TX 75390; 5 Neurology, University of Texas Southwestern Medical Center, Dallas, TX 75390;; 6 Uniformed Services University of Health Sciences (USUHS), Bethesda, MD 20814;; 7 Simmons Comprehensive Cancer Center, Dallas, TX 75390, USA

**Keywords:** prostate cancer, radioresistance, animal MRI, ultra-sound and image-guided radiation therapy

## Abstract

The purpose of this study was to develop an aggressive locally advanced orthotopic prostate cancer model for assessing high-dose image-guided radiation therapy combined with biological agents. For this study, we used a modified human prostate cancer (PCa) cell line, PC3, in which we knocked down a tumor suppressor protein, DAB2IP (PC3-KD). These prostate cancer cells were implanted into the prostate of nude or Copenhagen rats using either open surgical implantation or a minimally invasive procedure under ultrasound guidance. We report that: i) these DAB2IP-deficient PCa cells form a single focus of locally advanced aggressive tumors in both nude and Copenhagen rats; ii) the resulting tumors are highly aggressive and are poorly controlled after treatment with radiation alone; iii) ultrasound-guided tumor cell implantation can be used successfully for tumor development in the rat prostate; iv) precise measurement of the tumor volume and the treatment planning for radiation therapy can be obtained from ultrasound and MRI, respectively; and v) the use of a fiducial marker for enhanced radiotherapy localization in the rat orthotopic tumor. This model recapitulates radiation-resistant prostate cancers which can be used to demonstrate and quantify therapeutic response to combined modality treatments.

## Introduction

Prostate cancer is one of the most common male cancers, representing 28% of all male cancers in 2010 (1). In addition, prostate cancer was the second most common cause of cancer related death in US men (1). Treatment of prostate cancer can range from careful monitoring to treatment with prostatectomy or radiation. In most cases radical radiation therapy is very effective with 5 year local control rates of up to 89% (2). However, a proportion of patients will fail initial therapy and present with recurrent advanced local and distant metastatic disease. No curative treatment currently exists for those patients who present with recurrent disease.

In recent years, there has been a rise in the use of image guided radiation therapy (IGRT) in the treatment of prostate cancer. Because the prostate is a mobile organ, inter and intra fraction movement, as well as random deformation of the organ, can occur throughout the course of daily radiation treatment (3). Crevoisier *et al* showed that failure to compensate for daily prostate motion may lead to poorer clinical outcomes (4). Prostate position can be monitored through the use of fiducial markers that are visible on radiographic images. By using daily imaging it is possible to achieve accurate prostate localization and ensure homogeneous dose distributions.

While the use of image-guidance has improved the accuracy of radiotherapy, allowing safe and effective dose escalation in the treatment of primary prostate cancer, there is still no effective therapy for aggressive or recurrent disease. To develop effective therapies for aggressive prostate cancer, whether novel or a combination of several agents and treatment modalities, it is imperative to first develop a model of radiation resistant prostate cancer.

To facilitate the investigation of resistant aggressive disease after radiation in a preclinical environment it would be best to have a locally advanced orthotopic rodent model. In this study we report the development of such a model for resistant tumors after IGRT in rat prostates using a novel tumor suppressor knockdown prostate cancer cell line.

## Materials and methods

### Cell culture

Human PCa cell line PC3 was modified by knocking down the tumor suppressor protein DAB2IP (PC3-KD) and co-transfected with luciferase reporter gene as described previously by Kong *et al*(5). Cells were cultured in T medium supplemented with 5% fetal calf serum, 100 U/ml penicillin, 100 *μ*g/ml streptomycin, 900 *μ*g/ml of G418, and 700 ng/ml of puromycin in an atmosphere of 95% air/5% CO_2_ at 37°C.

### Orthotopic model

A total of 1×10^5^ PC3-KD cells were diluted to a final volume of 30 *μ*l. Male rats, either nude or Copenhagen, were anesthetized using 1–2% isoflurane mixed with 100% O_2_. Cells were injected into the right lobe of prostate and a gold fiducial marker was placed into the prostate for subsequent image guided therapy. All the experiments were conducted under UT Southwestern Institutional Animal Care and Use Committee-approved guidelines for animal welfare.

### Ultrasound

Ultrasound imaging was employed for i) minimally invasive implantation of tumor cells; ii) measuring tumor volume in the rat prostate. For implantation, rats were anesthetized and the pelvis was shaved and sterilized. Animals were secured to the handling table to ensure no movement. The prostate and bladder was identified in the field of view by using an RMV716 transducer head (VisualSonics Vevo^®^ 770 Imaging System, Amsterdam, The Netherlands). Once the prostate was visualized, a gold seed was implanted into the prostate using an 18G needle mounted on a trocar. The needle was aligned parallel to the transducer and inserted parallel to the urethra. After confirming proper placement of the fiducial, the needle was left in place and cells were implanted using a 1cc syringe. Imaging was performed on the VisualSonics Vevo 770 Imaging System using the real-time Micro Visualization scan head RMV716 (11–24 MHz) specific for rats. Rats were anesthetized and the transducer head was placed transverse to the pelvis of the rats in supine position. Images were obtained by resolving various depths of tissue into the center of optimum resolution plane to ensure clear images throughout the tumor volume.

### Bioluminescence imaging

BL imaging was performed weekly using an IVIS Lumina Imaging System (Xenogen, Alameda, CA). Rats were anesthetized by using isoflurane inhalation mixed in pure oxygen followed by an i.p. injection of D-luciferin (80 mg/kg). BL images were acquired 10 min after luciferin injection using various exposure times.

### Colony formation assay

Surviving fraction (SF) analysis was performed using PC3 Con (DAB2IP proficient) and PC3-KD (DAB2IP deficient) as described by Kong *et al*(5,6). In brief, cells were counted, serially diluted and plated in 60 mm dishes. After 6 h, treated with increasing doses of radiation (0 to 8 Gy) and then incubated 10 days for colony formation. Colonies were counted and SF curves were plotted using linear quadratic equation (Sigma plot 11.0, Systat Software, Inc).

### Magnetic resonance imaging

MRI studies were conducted using a 3 Tesla whole-body human scanner (Achieva™, Philips Medical Systems, Best, The Netherlands) with a small animal solenoid radio-frequency (RF) coil (63 mm in diameter and 100 mm in length; Philips Research Europe, Hamburg, Germany). Under anesthesia, animals were placed supine where the thigh of the rat centered with respect to the center of the RF coil. A volume containing the entire tumor was subsequently obtained using T2 weighted multi-slice fast spin echo sequences (repetition time, 5,700 msec; echo time, 70 msec; slice thickness, 2 mm; field of view, 75 × 48 × 50 mm in-plain resolution of 0.26 × 0.29 mm) and three dimensional gradient sequences (repetition time, 7.7 msec; echo time, 4.5 msec; flip angle 15, field of view = 75 × 51 × 50 mm resolution of 0.7 mm^3^ isotropic voxel).

### Microirradiation

Radiation was carried out using an X-ray image guided small animal irradiator as previously described (7,8). The irradiator is characterized by a high dose rate, small beam size, accurate and precise target localization facilitated through image guidance, resulting in precision-high dose irradiation. The collimation system consists of a 2.5-cm-thick brass alloy disk with interchangeable apertures ranging from 1 to 20 mm in nominal diameter.

### H&E staining

Prostate tumors were removed and fixed in 4% formalin. Tumors were mounted in paraffin and sections (10 *μ*m) were prepared for standard H&E staining. Briefly, sections were deparaffinized, rehydrated, stained with Harris’s hematoxylin for 20 sec followed by treatment with Scott’s solution. After washings, sections were stained with eosin for 2 min, rinsed, dehydrated in ethanol and xylenes and mounted using permount.

### Pimonidazole staining

Hypoxia staining in the rat prostate tumor was performed using the Hypoxyprobe™-1 plus kit (Hypoxyprobe Inc., Burlington, MA). Hypoxyprobe-1 (pimonidazole HCl) was administered i.p. (120 mg/kg) in tumor bearing rats. Two hours later, the animal was sacrificed; tumor tissue was collected and fixed in 4% formalin solution for 48 h. For detection, tumor sections were incubated with FITC-conjugated mouse monoclonal antibody against pimonidazole (1:50) for overnight at 4°C. After incubation with primary antibodies, tumor sections were washed thoroughly and visualized using a Zeiss Axio Imager 2 microscope (Carl Zeiss Microscopy, New York, NY) using the FITC filter.

## Results

### Development of an orthotopic prostate model for multimodal imaging

**Figure 1:**
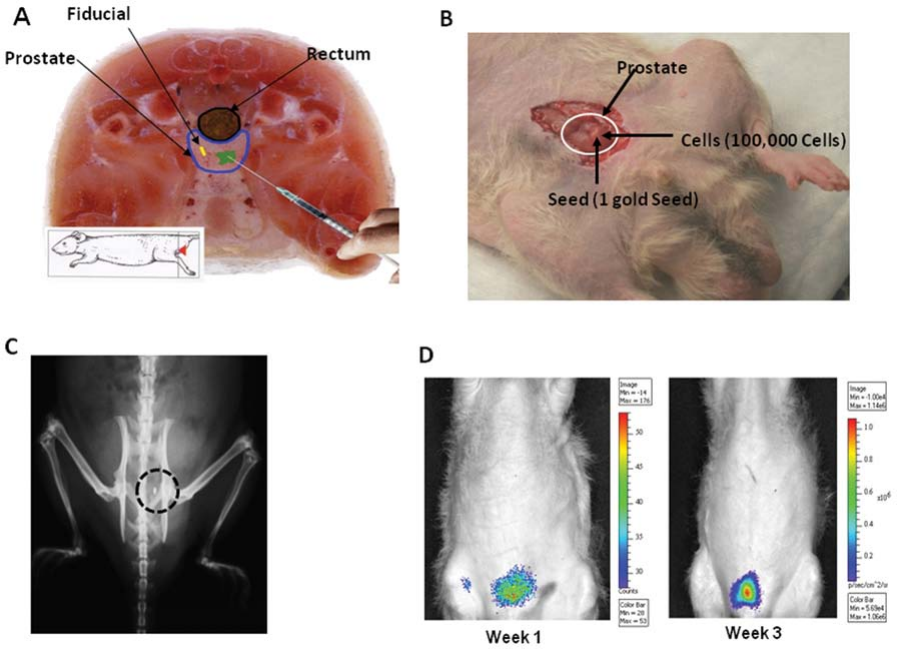
Orthotopic tumor generation in the rat prostate. (A) Transverse section of the rat anatomy showing the location of the prostate and rectum and schematic representation of the implantation of prostate cancer cells and fiducial (Courtesy of: A color atlas of sectional anatomy of rat; Toshiyuki Hayakawa and Takamasa Iwaki). (B) Tumor cells were placed using an open surgical method. The prostate was located by creating an incision into the abdomen and dissecting through the peritoneum. A gold fiducial marker was placed into the right lobe of the prostate using a trocar mounted on an 18 gauge needle and using the same needle, 1 ml syringe was attached and 1×10^5^ PC3-KD cells in 30 *μ*l were injected into the prostate. (C) X-ray of rat pelvis showing the gold fiducial successfully implanted into the prostate. (D) BLI confirming proper implantation of the tumor as well as to track growth.

To appropriately facilitate the study of IGRT for aggressive PCa, it is necessary to have a model that closely mimics human disease, ideally with a tumor that is initially limited to one lobe of the prostate. In this model, we implanted a human prostate tumor cell line which is deficient in a tumor suppressor protein DAB2IP (PC3-KD). This protein is a member of the Ras-GTPase activating family and the loss of DAB2IP has been associated with PI3K-Akt hyperactivation (9), increased radiation resistance (5,6), evasion of apoptosis (9), epithelial-mesenchymal transition and poor clinical outcomes (10). The PC3-KD cells were implanted either using an open surgical method in nude rats (Fig. 1A and B) or a minimally invasive method using ultrasound guidance in Copenhagen rats. Using the open surgical method cells were successfully implanted in all animals (n=17), however when using the ultrasound guided method the successful implantation rate was 70% (n=7). We also placed a gold fiducial marker as shown in Fig. 1C whereas, Fig. 1D shows BL imaging on weeks 1 and 3 after implantation.

**Figure 2:**
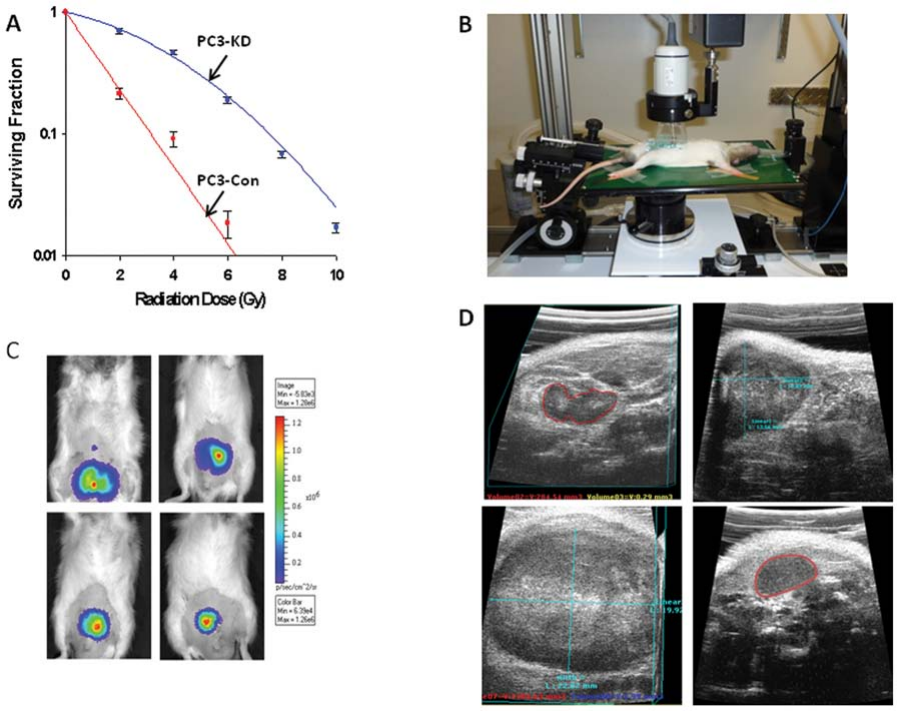
(A) Clonogenic survival assay using a PC3-Con (DAB2IP proficient) PC3-KD (DAB2IP silenced) cell line. (B) Set up of ultrasound imaging station for tumor cell implantation and imaging. (C) BLI of four different Copenhagen rats demonstrating various stages of tumor growth. (D) Ultrasound imaging of prostate as well as proximal pelvic organs. Prostate tumors are outlined in red. Each panel represents an axial ultrasound image of an OT tumor in the prostate. The left upper section of this image is notable for diffuse calcification and necrosis. Tumors reached diameters as large as 2 cm before being euthanized.

PC3-KD cells are highly aggressive and demonstrate significant radio-resistance (Fig. 2A). We also performed ultrasound guided PC3-KD cell implantation in Copenhagen rats (Fig. 2B) and then tumor progression was followed by BL imaging (Fig. 2C). Because of the poor inherent contrast between the prostate tissue and tumor, CT is not an optimal imaging modality for determination of size and location of prostate tumors. Therefore, we used ultrasound (Fig. 2D) and MRI to monitor tumor growth, size and location. By using ultrasound, it is possible to create three dimensional reconstructions of the tumor and track the tumor volume. Ultrasound images obtained from this model are notable for areas of necrosis and diffuse calcification throughout the tumor as represented by the varying echogeneity of the image (Fig. 2D).

**Figure 3:**
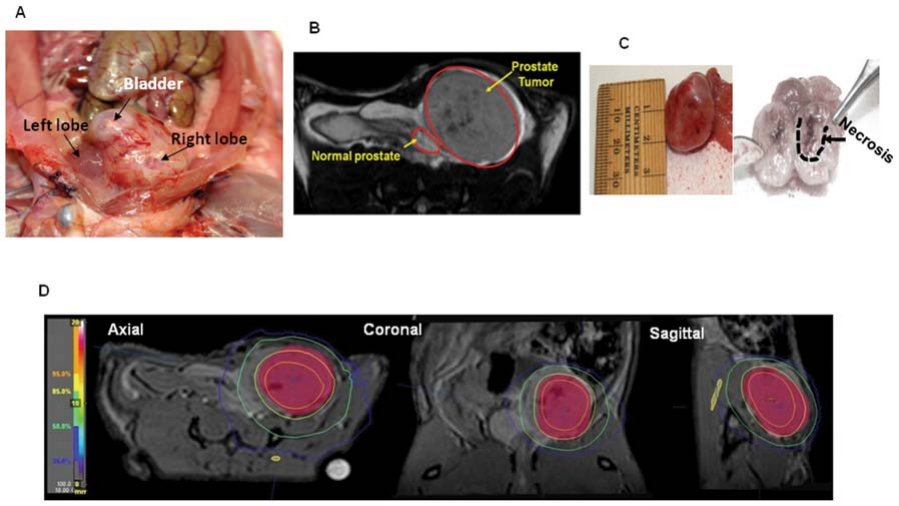
(A) Digital image displaying OT tumors in the rat pelvis after euthanasia. No visible metastasis was observed to other structures within the perineum or peritoneum. (B) MRI provides a non-invasive method to track tumor growth. (C) Specimen tumor resected en bloc with representative sizing. The tumor, once dissected, displays large grossly visible areas of necrosis. (D) MRI was used to create radiation treatment plans for the rats for applying uniform dose.

Fig. 3A displays the high resolution digital image of the OT tumors in the right prostate lobe of a nude rat. A specimen of such tumor was isolated from the prostate with representative sizing and dissection showing grossly visible areas of necrosis (Fig. 3C). We also performed MRI on nude rats (Fig. 3B) and this imaging modality in particular is highly useful for radiation treatment planning (Fig. 3D).

### Tumor growth and response to radiation therapy

**Figure 4:**
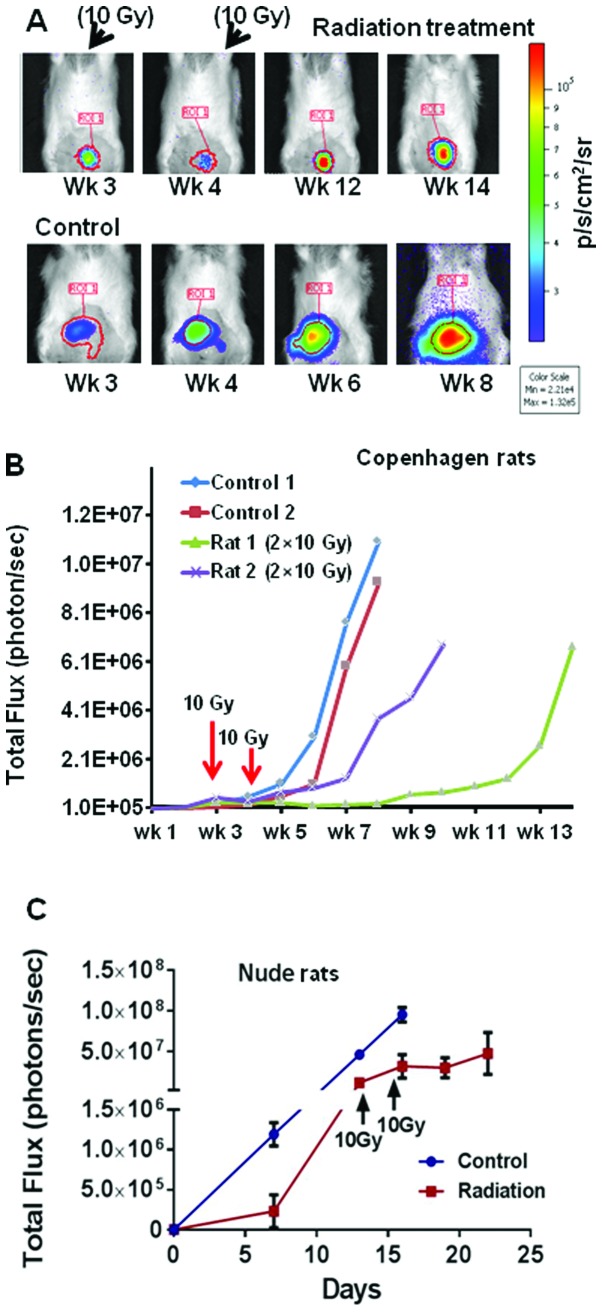
(A) The upper panel displays the tumor growth (rat 1) throughout the treatment course. The lower panel displays with the course of an untreated rat (control 1). The heat scale is given in signal intensity per unit area (p/s/cm^2^/sr). (B and C) Tumor growth curve (control and radiation treated) of the (B) Copenhagen rats and (C) Nude rats obtained by integrating the BLI signal (total Flux) over a region of interest. Arrows indicate the days when radiation was delivered.

PC3-KD cells were implanted into the prostate of Copenhagen (immune competent) and athymic nude rats (immune deficient). Tumor growth was followed by BLI as described above. Once the tumor size reached a certain size (approximately 5–7 mm in diameter) based on ultrasound imaging, animals were split into either a treatment (n=2 for Copenhagen; n=6 for Nude Rats, respectively) or control arm (n=2 for both arms). Fig. 4A (upper and lower panel) demonstrates tumor progression in Copenhagen rats after receiving radiation treatment.

Control group tumors demonstrated aggressive, but predictable growth (Fig. 4A, lower panel). We initiated radiation treatment on the rats when the total Flux (photon/sec) (integrated over an appropriate region of interest, ROI) reached approximately 2.6–3.0×10^5^ as shown in Fig. 4B. We allowed one Copenhagen rat to achieve a slightly larger tumor volume before initiating treatment (Fig. 4B). The rat was treated with 2 fractions of 10 Gy radiation as shown in Fig 4A. Radiation was delivered using image guidance to ensure that dose was delivered to the tumor. Though PC3-KD is an aggressive radioresistant cell line (Fig. 2A), the tumor demonstrated a significant initial response to radiation therapy (Fig. 4A and B). In Copenhagen rats it appears as if the tumors are initially controlled but begin to grow a few weeks after radiation. We noted that BLI signal does not completely resolve, remaining at a detectable level until week 7–8 (Fig. 4B). Between weeks 9–10, a new focus of intensity reappears at the site of the original tumor bed and ultimately grew uncontrollably and the animal died at the end of week 14 (Fig. 4B). The animal with a larger initial tumor (Rat 2) also received a similar dose (total 20 Gy) however, died earlier at week 11. Tumor burden led to compression of the urethra and partial obstruction of the rectum. Therefore, death was caused either by post renal failure or recto-sigmoid perforation secondary to fecalith impaction. However, it is clear that primary tumor caused the complication which led to death. It is also important to note that the peritoneum and mesentery were free of metastatic disease as observed in Fig. 3A. In contrast, rats receiving no radiation BLI signal increased continuously and the animals were euthanized at week 8 (Fig. 4A, lower panel, B).

The athymic nude rat tumors displayed the same aggressive growth pattern, however, they grew significantly faster than the tumors in Copenhagen rats. Control group displayed aggressive, but predictable growth; rats were euthanized on day 16. We initiated radiation as soon as signal appeared in the treatment rats (total Flux photons/sec; 5×10^5^ to 1×10^6^) (Fig. 4C). These rats were also treated with 2×10 Gy on days 13 and 16. Tumors display little response to radiation; ultimately tumor growth was delayed for a matter of days before resuming growth. By day 22 tumors were large enough to warrant euthanasia. Our previous mouse model study also showed that PC3-KD subcutaneous tumors are highly radioresistant when treated with fractionated radiation (6). These results clearly demonstrate that orthotopically implanted PC3-KD cells can recapitulate aggressive prostate tumors and furthermore, if not treated at an early stage, local control is difficult to achieve and this necessitates the use of pathway specific inhibitors in combination with radiation treatment. While possessing intrinsic radiation resistance, we demonstrate that DAB2IP deficient tumors can recur after initial response to appropriate IGRT.

**Figure 5:**
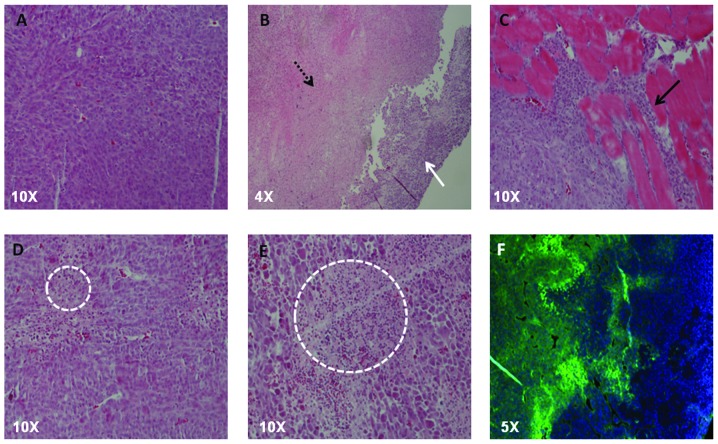
(A) H&E stained tissue section from a non-irradiated control; PC3-KD tumors are highly anaplastic and aggressive with high nuclear to cytoplasmic ratios, loss of cell polarity and loss of glandular structure. (B) H&E stained tissue section from an irradiated tumor. The irradiated area is highly necrotic (black dash arrow) displaying loss of cellular structure and high levels of eosin staining as compared to a strip of unirradiated tissue (white arrow). (C) H&E section of tumor showing invasion into adjacent skeletal muscle, the black arrow delineates tumor cells invading longitudinally down a skeletal muscle fascicle. (D) H&E section notable for areas of apoptosis. Apoptotic cells are noted for the loss of cellular detail as well as pyknotic nuclei (white dashed circle). (E) H&E image of radiated sections of tumor showing infiltration by neutrophils and macrophages in the early periods are radiation (white dashed circle), these areas will eventually become fibrotic. (F) Immunofluorescence using FITC conjugated antibodies against pimonidazole. Areas that are stained green represent areas of hypoxia, blue areas represent cell nuclei (DAPI). PC3-KD tumors show strong areas of central core hypoxia.

To correlate the imaging with biological events we performed IHC analysis. H&E stained sections confirm the placement of the tumor into the rat prostate; PC3-KD cells are highly anaplastic and aggressive (Fig. 5A). Orthotopically implanted tumors are very similar to human disease, tumors are locally aggressive as shown in Fig. 5C. Radiated rat prostates display gross necrosis and cell death, some areas of the tumor show changes indicative of apoptosis (Fig. 5B and D) these sections agree with the ultrasound findings which were indicative of necrosis. Changes seen in the irradiated rat prostates were consistent with reactive inflammation, neutrophils can be seen infiltrating the perivascular space as well as infiltrating the areas of necrosis (Fig. 5E). Using pimonidazole, we show that tumors develop large areas of hypoxia heterogeneously spread throughout the tumor (Fig. 5F). These areas of hypoxia may explain the high amount of resistance to radiation therapy.

## Discussion

While several other OT prostate tumors have been reported in the literature (11–13), there are currently no models that can accurately represent tumors that fail initial RT. With the increased use of genetic manipulation leading towards the addition of luciferase reporters to cell lines, it is now feasible to track tumor growth and response to treatment. Given that there is a lack of effective therapies for patients who present with biochemical failure, we felt that developing an aggressive locally advanced model that did not respond to radiation therapy alone was necessary to develop combined modality therapies capable of controlling aggressive tumors.

There are several requirements necessary for the development of a locally advanced prostate tumor specially for IGRT or combined modality therapy. Current OT prostate models for preclinical studies are primarily developed in mouse, however, to delineate tumors and track tumor response animals with larger prostates need to be used, hence we used rats. Secondly, the major difficulty in creating an OT model that accurately demonstrates human disease, specifically a tumor that is radiation resistant, is reliability. In order to facilitate resistance we used a DAB2IP knockdown prostate cell line. By using the DAB2IP knockdown cell line resistant tumors are reliably formed. Orthotopically implanted cells were able to grow large tumors in immune-competent male Copenhagen rats as well as nude rats.

Copenhagen rats are used primarily to study metastatic progression of prostate cancer as first described by Dunning (14–16). Several of the models developed to study prostate carcinoma did involve the injection of cells into the prostate (16), however, previous studies were done using syngeneic models which did not invoke a strong response when orthotopically implanted (17,18). Previous studies also investigated the radiation sensitivity of the Copenhagen rat prostate tumor model, which consists of anaplastic high grade tumors (15), and found that the tumors had radioresistant subpopulations *in vitro* but could not find correlating aggressive radiation resistant tumors *in vivo*(19). Furthermore, previous studies could not recapitulate recurrent disease (19).

It is very interesting to note the differences in growth rates between immune competent Copenhagen rats and athymic nude rats. Tumors in nude rats grow much quicker causing mass effect within days rather than weeks. Paradoxically, the rapidly growing nude rat tumors should be more radiation sensitive, however, our model shows that they are much more radiation resistant. It is possible that innate immunity, rather than humoral immunity, response of Copenhagen rats plays a significant role in controlling tumor proliferation. However, the Copenhagen study remains a pilot and this requires a larger more in depth study.

Once the tumors were successfully implanted they exhibited several characteristics pertinent to aggressive tumor growth. Radiation response also seems to correlate to initiation of treatment. In Copenhagen rats, treatment arm that received RT early regrowth is delayed by several weeks. However, the animal with the larger starting volume relapse was significantly shorter indicating that if treatment is delayed the tumor becomes more difficult to control. It is also important to note that based on the calculated α- and β-values of PC3-KD 2 fractions of 10 Gy leads to an LQED (Linear Quadratic Equivalent Dose) 2 Gy of 60 Gy, a dose that is clinically relevant in the treatment of human PCa. Furthermore, rapidly growing tumors often display heterogeneous areas of necrosis as a result of insufficient vascular supply (20). Insufficient blood supply leads to hypoxia which correlates to poor response. Ultrasound imaging of large tumors demonstrate large areas of necrosis as well as diffuse calcification and pimonidazole staining confirms that implanted tumors rapidly develop several large hypoxic areas.

In radiation resistant models, the ability to track tumor growth and response to therapy is essential. BLI was the primary imaging modality in this study and has been correlated with both CT as well as MRI (21). We further evaluated our model through the use of ultrasound. Here we demonstrate that ultrasound technology can be used successfully for the determination of tumor volume as well as to aid in tumor cell implantation. Ultrasound was also helpful in revealing additional information regarding the accurate localization, calcification, necrosis and the effects of the tumor on proximal pelvic organs such as bladder, which are not appreciable on BLI. While both imaging modalities could be used individually, the complementary information provided using both modalities creates a complete image of the tumor.
